# Development of the Multidimensional Readiness and Enablement Index for Health Technology (READHY) Tool to Measure Individuals’ Health Technology Readiness: Initial Testing in a Cancer Rehabilitation Setting

**DOI:** 10.2196/10377

**Published:** 2019-02-12

**Authors:** Lars Kayser, Sine Rossen, Astrid Karnoe, Gerald Elsworth, Jette Vibe-Petersen, Jesper Frank Christensen, Mathias Ried-Larsen, Richard H Osborne

**Affiliations:** 1 Department of Public Health University of Copenhagen Copenhagen Denmark; 2 Centre of Inflammation and Metabolism Rigshospitalet Copenhagen Denmark; 3 Centre for Physical Activity Research Rigshospitalet Copenhagen Denmark; 4 Danish Multiple Sclerosis Society Valby Denmark; 5 Centre for Population Health Research Faculty of Health Deakin University Geelong Australia; 6 Copenhagen Centre for Cancer and Health Municipality of Copenhagen Copenhagen Denmark

**Keywords:** health technology readiness, questionnaire, eHealth literacy, enablement

## Abstract

**Background:**

The increasing digitization of health care services with enhanced access to fast internet connections, along with wide use of smartphones, offers the opportunity to get health advice or treatment remotely. For service providers, it is important to consider how consumers can take full advantage of available services and how this can create an enabling environment. However, it is important to consider the digital context and the attributes of current and future users, such as their readiness (ie, knowledge, skills, and attitudes, including trust and motivation).

**Objective:**

The objective of this study was to evaluate how the eHealth Literacy Questionnaire (eHLQ) combined with selected dimensions from the Health Education Impact Questionnaire (heiQ) and the Health Literacy Questionnaire (HLQ) can be used together as an instrument to characterize an individual’s level of health technology readiness and explore how the generated data can be used to create health technology readiness profiles of potential users of health technologies and digital health services.

**Methods:**

We administered the instrument and sociodemographic questions to a population of 305 patients with a recent cancer diagnosis referred to rehabilitation in a setting that plans to introduce various technologies to assist the individuals. We evaluated properties of the Readiness and Enablement Index for Health Technology (READHY) instrument using confirmatory factor analysis, convergent and discriminant validity analysis, and exploratory factor analysis. To identify different health technology readiness profiles in the population, we further analyzed the data using hierarchical and k-means cluster analysis.

**Results:**

The confirmatory factor analysis found a suitable fit for the 13 factors with only 1 cross-loading of 1 item between 2 dimensions. The convergent and discriminant validity analysis revealed many factor correlations, suggesting that, in this population, a more parsimonious model might be achieved. Exploratory factor analysis pointed to 5 to 6 constructs based on aggregates of the existing dimensions. The results were not satisfactory, so we performed an 8-factor confirmatory factor analysis, resulting in a good fit with only 1 item cross-loading between 2 dimensions. Cluster analysis showed that data from the READHY instrument can be clustered to create meaningful health technology readiness profiles of users.

**Conclusions:**

The 13 dimensions from heiQ, HLQ, and eHLQ can be used in combination to describe a user’s health technology readiness level and degree of enablement. Further studies in other populations are needed to understand whether the associations between dimensions are consistent and the number of dimensions can be reduced.

## Introduction

### Background

The modernization of health care systems and the introduction of technologies are changing how health care systems are being improved and designed, not only in regard to quality and safety, but also for reach and active reduction of health inequalities. To achieve the potential benefits of digitization involving patients, it is important to address the individual’s digital readiness. Here, we define readiness in accordance with the Oxford dictionary [1] as *being prepared and willing*, where prepared is interpreted as the result of an individual’s knowledge, skills, and attitudes, including trust and motivation, which should be considered as enabling factors as they expand an individual’s possibilities to act [2].

Increasing digitization may benefit users and bridge geographical or social gaps [3]. Digitization may, however, impose new barriers for those with low literacy, who are reluctant to use technology [4,5] or have limitations due to physical or cognitive disorders, or who don’t have the requisite skills and attitudes, including trust and motivation (ie, those who are not prepared and willing). Health technology has the potential to support those who have the resources and competence to take advantage of the ever-expanding market of public and private health services, and has the potential to identify, include, and empower those who are disadvantaged and may be underserved, if these barriers can be overcome. It is important to be able to differentiate between users with respect to their needs, resources, and health technology readiness in order to be able to provide stratified solutions that address these user differences [6]. In a recent systematic review on instruments generally used to evaluate eHealth interventions, Wakefield et al identified a need for psychometrically robust instruments that are concept based; include several aspects of use, including eHealth literacy; and can be used across platforms and technology [7]. In their viewpoint on eHealth literacy, Griebel et al called attention to a lack of “a well-founded theoretical basis and approaches to put eHealth literacy in a broader context” and “how to link measured levels of eHealth literacy to the development of eHealth services” [8].

To be able to effectively discriminate between users, there is an imperative to fully conceptualize and measure health technology readiness of users or potential consumers of technology-assisted health care services. Despite that a recent review identified 8 frameworks for eHealth readiness [9], we have not been able to identify robust, psychometrically sound instruments to assess eHealth or health technology readiness. The only 2 instruments we could identify were the Patient eHealth Readiness Questionnaire (PERQ) and the Service User Technology Acceptability Questionnaire (SUTAQ). PERQ is not conceptually based or a psychometric instrument but consists of specific questions in relation to internet use, social support, personal abilities, and economic barriers [10]. SUTAQ was developed to assess technology acceptance in the Whole System Demonstrator project and is conceptually and psychometrically robust. The context is the user’s experience with a given technology, where an experience of reduced privacy and comfort predicts rejection of the technology, whereas an experience of benefits by the user lowers the likelihood of rejection [11].

### Proposal for the Readiness and Enablement Index for Health Technology

Here, we propose a concept-based, psychometrically sound, validated instrument, the Readiness and Enablement Index for Health Technology (READHY), based on the concept of eHealth literacy supplemented with relevant scales from other instruments assessing aspects of self-management and social support: the eHealth Literacy Questionnaire (eHLQ) [12], the Health Education Impact Questionnaire (heiQ) [13], and the Health Literacy Questionnaire (HLQ) [14].

In the period of 2015 to 2017, we developed the eHLQ [12] based on our previously published eHealth literacy framework [15] to enable systematic assessment of eHealth literacy. The eHLQ describes the user’s knowledge and skills, and their interaction and experiences with digital health services and technology. The dimensions are as follows: (1) using technology to process health information, (2) understanding of health concepts and language, (3) ability to actively engage with digital services, (4) feel safe and in control, (5) motivated to engage with digital services, (6) access to digital services that work, and (7) digital services that suit individual needs. The eHLQ provides the means to understand an individual’s health technology readiness by addressing dimensions of knowledge and skills (eHLQ scales 1 to 3), user experiences (eHLQ6 and eHLQ7), and user trust (eHLQ4) and motivation (eHLQ5). However, Gilstad pointed out that eHealth literacy needs to be understood in a cultural, social, and institutional context [16], and May et al proposed that the burden of treatment may have a negative influence on the individual’s mental state [17]. We recognize that these additional areas may be important to assist with fully understanding the degree to which individuals are able, prepared, and willing to use health technologies; therefore, supplementing the eHLQ with these additional areas should deepen our understanding of end users’ readiness for full participation in health technology and to monitor how health technology potentially enable users.

We therefore decided to evaluate whether dimensions from the heiQ, which assesses self-management, and domains from the HLQ, which assesses health literacy, including support from relatives, peers, and health professionals, could supplement the eHLQ. The heiQ comprehensibly evaluates the impact of health education interventions on self-management [13]. The heiQ has 8 dimensions: (1) health-directed activities, (2) positive and active engagement in life, (3) self-monitoring and insight, (4) constructive attitudes and approaches, (5) skill and technique acquisition, (6) social integration and support, (7) health services navigation, and (8) emotional distress. We considered the HLQ, as 2 of its 9 dimensions include the aspect of social context. The HLQ consists of 9 dimensions: (1) feeling understood and supported by health care providers, (2) having sufficient information to manage my health, (3) actively managing my health, (4) social support for health, (5) appraisal of health information, (6) ability to actively engage health care providers, (7) navigating the health care system, (8) ability to find good health information, and (9) understanding health information well enough to know what to do. All 3 instruments were developed using a concept mapping process and a validity-driven approach [18], in which all items are based on statements from users and clustered into concepts that are grounded in the users’ collective experiences and knowledge.

**Figure 1:**
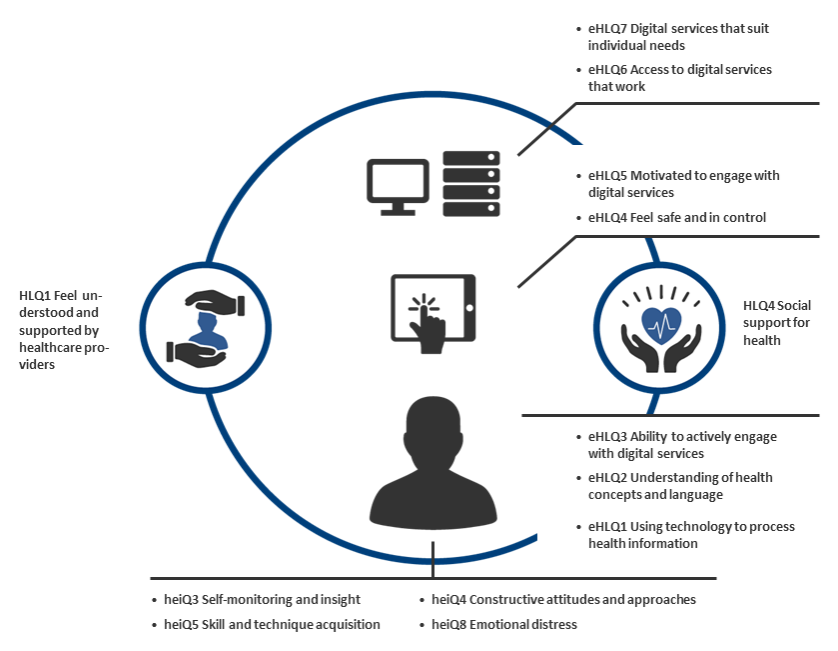
The 13 dimensions of the Readiness and Enablement Index for Health Technology (READHY) (modified from [12]). The 7 eHealth Literacy Questionnaire (eHLQ) dimensions describe users’ attributes; the intersection between users and technologies; and users’ experience of systems. The 4 Health Education Impact Questionnaire (heiQ) dimensions add knowledge about the individuals’ capabilities to handle their condition and emotional response. The 2 eHLQ dimensions add knowledge about individuals’ social context (represented by the circle encompassing the individual and the individual’s attributes).

To identify suitable dimensions from the heiQ and HLQ, covering social context, capabilities to handle the situation, and burden of disease and treatment, to add to the eHLQ to obtain a complete health technology readiness instrument, we (LK and AK) mapped the 24 dimensions of the eHLQ, heiQ, and HLQ and evaluated the content of the items for this purpose. In 2016, we identified possible dimensions to be included and used information from the literature and ongoing projects known to us. We identified 13 dimensions (Figure 1) from the 3 conceptually distinct instruments. The 7 eHLQ dimensions describe the attributes of the users (information and knowledge about their health and use of technology); the intersection between users and the technologies (their feeling of being safe and in control and their motivation); and users’ experience of systems (they work, are accessible, and suits users’ needs) [12]. From the heiQ, we selected heiQ3 (self-monitoring and insight), heiQ4 (constructive attitudes and approaches), heiQ5 (skill and technique acquisition), and heiQ8 (emotional distress), as evidence was available that they reflect intended outcomes relevant to educational or technological interventions (Multimedia Appendix 1). These candidate dimensions reflect an individual’s capabilities to handle their condition and emotional response. From HLQ, we selected HLQ1 (feeling understood and supported by health care providers) and HLQ4 (social support for health), as they add knowledge about the interaction with and impact of social and health care provider networks.

### Objective

The aim of this study was to evaluate this new unified instrument, READHY, with exploratory factor analysis (EFA) and confirmatory factor analysis (CFA) and to suggest how the generated data can be used to assess the readiness of potential users of health technologies and digital health services, as well as their degree of enablement; that is, users’ knowledge and skills, their self-management of disease, their perceptions and mindset, their experience with health technology systems, and an understanding of the extent to which users feel supported by relatives, peers, and health professionals.

## Methods

### Research Design, Setting, and Test Population

This study was part of a larger study investigating health technology readiness and motivation for training in the Copenhagen Centre for Cancer and Health, Copenhagen, Denmark. The study used a cross-sectional design with convenience sampling among patients referred to cancer rehabilitation in the Centre in the period August 2016 to July 2017. Questionnaires were administered in the period October 2016 to July 2017. We selected this setting because the organization is legally obligated to offer cancer rehabilitation and is interested in the development of a digital intervention for this context. Patients were either asked to participate in connection with their assessment consultation with their Centre contact person or contacted by telephone by a research project member.

Exclusion criteria were age less than 18 years, insufficient cognitive function, or inability to understand Danish. Individuals who could not be contacted by telephone were excluded after 3 attempts to reach them at varying times. The patient’s contact person also had the opportunity to decline a potential participant’s participation in the study for reasons not stated.

We considered 300 an adequate sample size for the CFA [19-21]. For example, Iacobucci [19], in reviewing the effect of sample size on convergence, numerical quality, and the typical fit indices used in CFA (and which are also available for EFA and exploratory structural equation modeling [ESEM] in Mplus), concluded that “It is of some comfort that SEM models can perform well, even with small samples (e.g., 50 to 100).” (pg 92). Similarly, Bandalos [20] and Forero et al [21] quoted studies where diagonally weighted least squares analysis (as used in our investigation in the form of weighted least squares means and variance adjusted [WLSMV] in Mplus) performed acceptably at sample sizes larger than 200, while their own simulation studies suggested that, while larger sample sizes provide increasingly robust results, samples of approximately 300 typically produce relatively unbiased parameter estimates and standard errors with diagonally weighted least squares.

All participants answered a background information questionnaire, the Behavioral Regulation in Exercise Questionnaire, and the READHY instrument.

### The READHY Instrument

The READHY instrument consists of 13 dimensions with a total of 65 items from the heiQ, HLQ, and eHLQ. All 13 scales reflect the conceptual dimensions and are rated on a Likert-type scale from 1 (strongly disagree) to 4 (strongly agree). The participants filled out the instrument in paper form. If the participant wished, a research project member was present to clarify questions or assist the participant. The READHY instrument was administered in the same order to all participants. The items were grouped according to the instrument they belong to and administered in the same sequence as in the original instruments. We calculated the overall score of each dimension as the mean of the 4 to 6 items constituting the dimension. Regarding missing items, if 50% or more of the items in a dimension were answered, we calculated an average for the dimension based on the filled-in items.

### Statistics

#### Evaluation of the Properties of READHY Using Confirmatory Factor Analysis

We based factor analysis of the selected 13 heiQ, HLQ, and eHLQ scales on a sequence that followed and extended the general idea of “semiconfirmatory” factor analysis suggested by McDonald [22]. A semiconfirmatory factor analysis is partially confirmatory and partially exploratory. Its aim is to use a known set of homogeneous independent clusters of items as the basis for the exploration of the location of further items that are less well understood in relation to these established item clusters ([22], pg 165). In our case, we considered the 7 eHLQ dimensions as the starting basis for the analysis and aimed to explore the relationship of the selected additional heiQ and HLQ scales to this basic structure. We were, however, cognizant that some eHLQ scales are quite highly correlated [12] and may not be clearly distinguishable in a combined-factor structure. To this end, we incorporated an analysis of the convergent and discriminant validity of the 13 selected scales into the procedure to establish whether some might be combined in a more parsimonious integrated structure. We conducted all analyses with Mplus 8 (Muthén & Muthén), mostly using the ESEM program feature with polychoric correlations, WLSMV estimation appropriate for ordinal data, and geomin rotation when we applied EFA.

The sequence of analyses was as follows. (1) The first analysis was a CFA hypothesizing 13 factors, followed by an analysis of the convergent and discriminant validity of the 13 factors based on the CFA results, using Fornell and Larcker’s criteria [23,24]. If the analysis of convergent and discriminant validity suggested that some of the scales were showing insufficient discriminant validity, a more parsimonious factor structure might yield a satisfactory fit to the data. (2) The CFA and analysis of convergent and discriminant validity was followed by a full EFA. When a parallel analysis and scree slope from this analysis clearly suggested that a more parsimonious factor solution might be a satisfactory account of the relationships between the 13 scales, we applied a series of EFA analyses to extract a smaller number of factors. (3) We then hypothesized an ESEM model based on the previous analyses. (4) A final CFA to confirm the revised factor structure and estimate its fit to the data completed the sequence.

We assessed model goodness-of-fit by the root mean square error of approximation (RMSEA), comparative fit index (CFI), and Tucker-Lewis fit index (TLI) [25]. We regard a well-fitting model as one where the RMSEA is less than .06 and the CFI and TLI are greater than .95, while we took a value of less than .08 for the RMSEA to indicate a reasonable fit [26-29].

#### Cluster Analysis

The READHY instrument is intended to characterize populations stratified by their level of health technology readiness. Inspired by the creation of personas in information technology systems development [30] and by the recently published model for the Optimising Health Literacy and Access (OPHELIA) process [31,32], we explored how dimensions can be used for modeling profiles or data to develop personas. To make interpretation easier, we reversed the scale heiQ8 (emotional distress) so that a high score meant less distress.

We applied a combined approach using hierarchical analysis to perform an exploratory evaluation and then the k-means method to evaluate the strength of the resulting number of clusters and to characterize the subgroups. We performed cluster analysis using IBM SPSS statistics version 22 (IBM Corporation). To identify the appropriate number of clusters, we used the method from the OPHELIA process; we performed the hierarchical approach using Ward’s method for linkage [33] for a range of cluster solutions. The appropriate number of clusters for the dataset was guided by examining the agglomeration schedule to identify the demarcation point (Multimedia Appendix 2) and the dendrogram to identify when the variance of the dimensions within the clusters increased. We also compared the standard deviations for the group mean profiles of the different cluster solutions, as standard deviations greater than 0.6 could indicate that there are still significant subgroups within the cluster [32]. Guided by the findings of the hierarchical cluster analysis, we performed a k-means cluster analysis of the dataset. As opposed to hierarchical clustering methods where cases are consecutively added to existing clusters, the k-means algorithm constantly reassigns cases to clusters independently of former assignments to minimize the within-cluster variation [34,35]. To obtain a meaningful number of clusters in the given context of cancer survivors, we performed a range of k-means cluster (2-8) solutions, lying around the demarcation point of the hierarchical analysis, to identify the best fit. We evaluated the appropriateness of the k-means cluster solution by the number of dimensions with a standard deviation greater than 0.6 and by examining the 1-way analysis of variance performed for each dimension to see whether there where variables not contributing to cluster separation (insignificant *F* values) [35]. We populated the various cluster solutions with sociodemographic data to understand which number of clusters made the most sense (from the clinical and sociodemographic sense) in the context of cancer survivors receiving rehabilitation. The final number of clusters decided on should reflect the given population and for what purpose the clusters will be used. In our context of cancer survivors receiving rehabilitation, possibly involving technology, we found a 4-cluster solution to be the most suitable for stratification, when taking both the statistical results and the sociodemographic data into consideration (S Rossen MSc PhD, unpublished data, 2018). We also present an 8-cluster solution to illustrate how clustering can be used for other purposes in explaining and identifying particularly vulnerable subgroups and their characteristics.

### Ethics

The project complied with the Declaration of Helsinki and was approved by the Danish Data Protection Agency (2015-55-0630). Under Danish law, permission from an ethics committee was not required because biological material was not used in the study. All participants received oral and written information about the survey and were informed that their participation was voluntary, that they were ensured anonymity, and that all data would be handled confidentially. We obtained written informed consent from all participants.

## Results

### Study Participants

**Figure 2:**
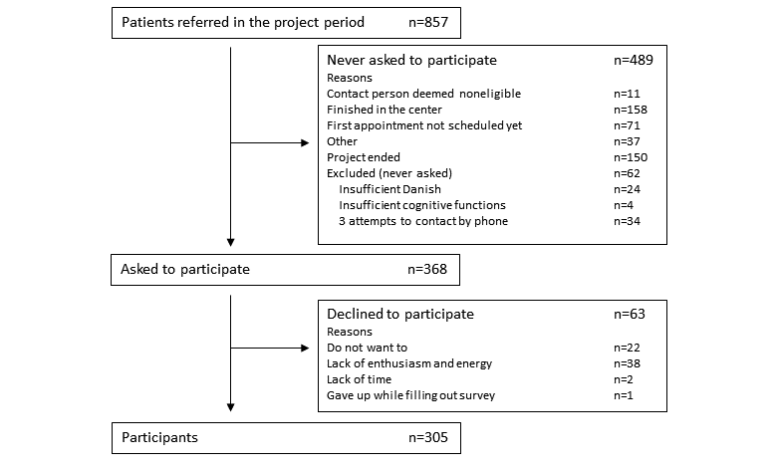
Flowchart of the study population.

Of 857 patients referred in the project period, 368 were asked to participate. Of these, 63 declined to participate, resulting in 305 participants (see Figure 2 for study population flowchart). Participants had a mean age of 58 years (ranging from 18 to 90 years), 70.8% (216/305) were women, and 81.6% (249/305) owned a smartphone.

### Evaluation of the Properties of READHY Using Confirmatory Factor Analysis

The 13-factor CFA demonstrated a reasonable fit for the 13 factors (RMSEA=.049, 95% CI 0.046-0.052; CFI=.94; TLI=.935). With the exception of heiQ3 and eHLQ6, all scales were well identified by the hypothesized items. Only item 10 cross-loaded between heiQ3 and heiQ8 (see Multimedia Appendix 3).

For 2 scales, heiQ3 and eHLQ6, the average variance extracted was less than .5 (Fornell and Larcker’s first criterion [23]) and thus where convergent validity was questionable (Multimedia Appendix 4). Also, following Fornell and Larcker’s second criterion, 9 pairs of scales showed insufficient discriminant validity where the average variance extracted of 1 or the other in the pair was less than the variance shared between the 2 (heiQ3 and heiQ5; heiQ3 and eHLQ2; eHLQ1 and eHLQ5; eHLQ1 and eHLQ 6; eHLQ2 and eHLQ6; eHLQ3 and eHLQ6; eHLQ5 and eHLQ6; eHLQ5 and eHLQ7; eHLQ and eHLQ7). eHLQ4 showed good discriminant validity, while eHLQ2 was problematic in relation to eHLQ6 because of the low convergent validity of eHLQ6.

This suggests that, in this population, a more parsimonious model with a fit similar to the 13-factor model might be achieved. Therefore, we performed, an EFA. The parallel analysis, conducted with maximum likelihood estimation in Mplus, clearly suggested a 5-factor solution, while the scree slope plotted from the eigenvalues of the polychoric correlations used in the WLSMV analyses suggested that a 5- or 6-factor solution might be suitable (Multimedia Appendix 5). When we used close-fit criteria, the 6-factor solution was a satisfactory fit to the data (RMSEA=.046, 95% CI 0.043-0.049; CFI=.953; TLI=.943) with both the RMSEA and the CFI satisfying the prespecified cutoff values, whereas the fit of the 5-factor solution was less satisfactory (RMSEA=.052, 95% CI 0.049-0.055; CFI=.938; TLI=.927). The factor loading pattern from this solution generally paralleled the results of the convergent and discriminant analysis of the 13-factor CFA solution. Items from heiQ scales 4 and 8 loaded on 1 bipolar factor that contrasted the 2 constructs, while items from heiQ scales 3, 4, and 5 loaded on another factor. One resolution of this pattern would be to hypothesize 3 discrete heiQ factors consisting of heiQ4 items, heiQ8 items, and items from heiQ3 and heiQ5 combined. Items from HLQ1 and HLQ4 loaded on the same factor, while another factor was constituted only by secondary loadings from HLQ4 items. The eHLQ items separated into 1 large factor and a smaller factor constituted by eHLQ4, some eHLQ6 items, and, secondarily, 2 eHLQ7 items.

Given the lack of clarity in this solution, we extended the EFA analyses to 7 and 8 factors, and subsequently an 8-factor ESEM model. The ESEM model posited 3 factors comprising heiQ scales (heiQ4 and heiQ8 separately, and heiQ3 and heiQ5 combined) and 2 HLQ scales (HLQ1 and HLQ4 separately). These were CFA factors in that cross-loadings between them were not allowed. Additionally, the model posited 3 factors comprising eHLQ items that were fitted by an EFA component with geomin rotation. The fit of this 8-factor ESEM model was good by all close-fit criteria (RMSEA=.043, 95% CI 0.040-0.046; CFI=.953; TLI=.950). This model clearly confirmed heiQ4, heiQ8, and heiQ3/heiQ5 combined, and HLQ1 and HLQ4 as independent factors. It also suggested that eHLQ1, eHLQ3, eHLQ5, eHLQ6, and eHLQ7 items largely combined into 1 factor with items from eHLQ2 and eHLQ4 identifying separate factors. We conducted a final CFA analysis to confirm this 8-factor model (Multimedia Appendix 6). We required all items to load on only 1 factor as suggested by the ESEM analysis, with 1 exception: we allowed eHLQ item 33 to load on both the “omnibus” eHLQ factor and the eHLQ factor comprising eHLQ4 items. Fit was satisfactory given the extensive model constraints of only 1 allowed cross-loading and no residual correlations (RMSEA=.053, 95% CI 0.051-0.056; CFI=.927; TLI=.923). All hypothesized loadings, with 3 exceptions (including item 33), were greater than 0.4, and all but 6 loadings were greater than 0.5. Item 33, originally an eHLQ6 item was, in this sample, clearly associated with eHLQ4 items.

### Cluster Analysis

**Figure 3:**
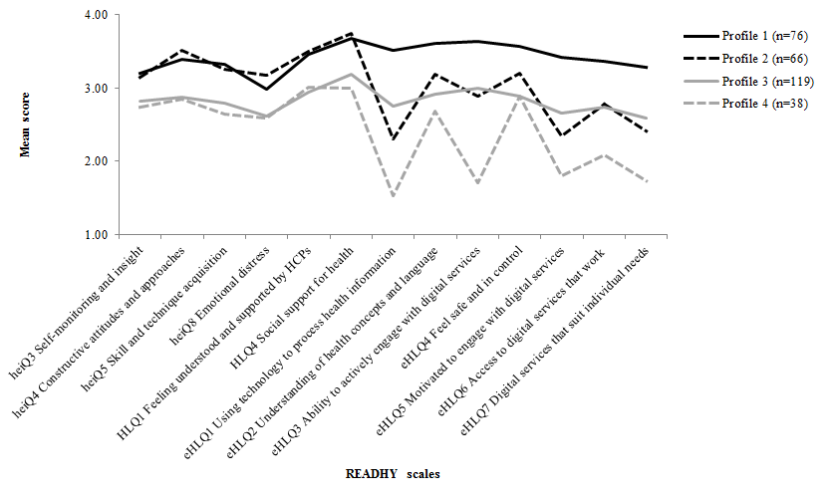
Four health technology readiness profiles based on cluster analysis of questionnaires administered to 305 people with a recent diagnosis of cancer. Health Education Impact Questionnaire (heiQ) dimension 8 was reverse scored so that a high score means a low level of distress. eHLQ: eHealth Literacy Questionnaire; HCPs: health care providers; HLQ: Health Literacy Questionnaire; READHY: Readiness and Enablement Index for Health Technology.

The hierarchical cluster analysis suggested a 4-cluster solution to be appropriate, and we explored the characteristics of 3, 4, and 5 k-means cluster solutions. Based on the k-means evaluation, we concluded that in this dataset a 4-cluster solution was the best fit. The magnitude of the *F* values from the analysis of variance performed on each dimension indicated that, in particular, eHLQ dimensions discriminated between the clusters. The subgroups of the 4-cluster solution were distinct in their READHY profiles (Figure 3). The heiQ and HLQ scales distinguished between profiles 1 and 2 (high) on the one hand and profiles 3 and 4 (lower) on the other. The eHLQ scales identified 3 groups: profile 1 (high on all), profiles 2 and 3 (middle on all), and profile 4 (low on 5). Profile 4 was lowest on the 5 scales that seemed to be most clearly measuring motivation, access, and capability with digital health services. Profile 1 had the highest smartphone ownership, was the youngest, and had the lowest number of chronic conditions. Profile 4, with the lowest scores, was the oldest and least likely to own a smartphone (Table 1).

**Figure 4:**
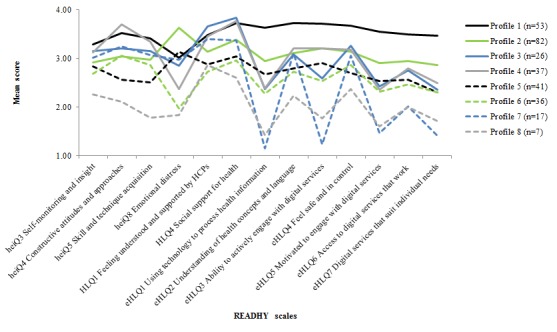
Eight health technology readiness profiles based on cluster analysis of questionnaires administered to 305 people with a recent diagnosis of cancer. Health Education Impact Questionnaire (heiQ) dimension 8 was reverse scored so that a high score means low level of distress. eHLQ: eHealth Literacy Questionnaire; HCPs: health care providers; HLQ: Health Literacy Questionnaire; READHY: Readiness and Enablement Index for Health Technology.

When we conducted clustering to identify individuals belonging to certain subgroups, an 8-cluster solution (Figure 4) revealed 2 profiles, 6 and 8, that scored low in the heiQ scales compared with the other clusters. Profile 8 was low on HLQ4 (social support for health) and all eHLQ scales, while profile 6 was in the middle range on HLQ and eHLQ scales. Profile 7 was very low on 4 of the 5 eHLQ scales associated most directly with eHealth motivation, access, and capability and middle to relatively high on heiQ and HLQ scales. Profile 1 was high in eHLQ scales and relatively high in the heiQ and HLQ scales. Profile 8 had a higher number of chronic conditions and was least likely to own a smartphone (Table 1). Profile 6 had a high smartphone ownership and an average of 1 additional chronic condition. Profile 7 was the oldest, had the highest number of chronic conditions, and was less likely to have a smartphone. Profile 1 was the youngest, had the lowest number of chronic conditions, and had the highest smartphone ownership.

**Table 1:** Demographic characteristics of individuals across 4- and 8-cluster health technology readiness profiles.

Profiles		Age (years), mean (SD)	Own a smartphone, n (%)	Number of chronic conditions (other than cancer), mean (SD)
**4-cluster**				
	Profile 1 (n=76)	53.9 (13.3)	70 (92)	0.59 (0.87)
	Profile 2 (n=66)	58.1 (14.7)	52 (79)	0.67 (0.84)
	Profile 3 (n=119)	58.1 (12.8)	106 (89)	0.73 (0.88)
	Profile 4 (n=38)	67.8 (12.5)	16 (42)	1.34 (1.05)
**8-cluster**				
	Profile 1 (n=53)	55.1 (13.7)	49 (92)	0.58 (0.93)
	Profile 2 (n=37)	56.0 (15.5)	33 (89)	0.67 (0.93)
	Profile 3 (n=82)	57.2 (13.2)	72 (88)	0.70 (0.80)
	Profile 4 (n=26)	61.2 (12.7)	17 (65)	0.76 (0.97)
	Profile 5 (n=36)	62.8 (12.4)	26 (72)	0.56 (0.65)
	Profile 6 (n=41)	55.3 (13.9)	36 (88)	1.05 (1.02)
	Profile 7 (n=17)	70.9 (10.1)	9 (53)	1.35 (1.17)
	Profile 8 (n=7)	58.9 (15.1)	2 (29)	1.29 (0.95)

## Discussion

### Health Technology Readiness and Enablement

This study presents a new way to understand user health technology readiness by taking advantage of the recently developed eHLQ instrument, supplemented with scales from other psychometrically sound instruments, to create the READHY instrument. The READHY covers aspects of users’ knowledge and skills, their self-management of disease, their perceptions and mindset, their experience with health technology systems, and an understanding of the extent to which users feel supported by relatives, peers, and health professionals. Understanding of technology readiness is usually associated with the maturity of a technology [36], including the organizational context [37]. READHY addresses the complementary situation: how ready and able the user is to engage with and take advantage of technologies. This adds to the understanding of the complex situation of a user’s interaction with digital tools in a health context.

The eHLQ is a multidimensional instrument that measures eHealth literacy strengths and weaknesses. Each dimension is represented by an independent scale, and the dimensions collectively provide a comprehensive profile of the informant. The eHLQ can be used alone in digital or technology contexts [12]. By adding scales from 2 other psychometrically sound and well-tested instruments derived from the lived experience of users, we extend the utility of the eHLQ to generate a comprehensive description of health technology readiness, as well as ancillary elements that capture the degree to which they are enabled: that is, determinants of users’ technological capability, their emotional distress, and their ability to manage their own condition over time. Technology-based interventions may also change the way the individual interacts with health professionals, peers, and relatives. Whether enablement is related to a higher or lower score on the HLQ scales (HLQ1, feeling understood and supported by health care providers; HLQ4, social support for health) remains to be explored.

The strength of the READHY instrument is that the eHLQ dimensions provide insight into users’ knowledge and skills (eHLQ1, using technology to process health information; eHLQ2, understanding of health concepts and language; eHLQ3, ability to actively engage with digital services), their self-management of disease (heiQ3, self-monitoring and insight; heiQ5, skills and technique acquisition), their perceptions and mindset (eHLQ4, feel safe and in control; eHLQ5, motivated to engage with digital services; heiQ4, constructive attitudes and approaches; heiQ8, emotional distress), their experience with health technology systems (eHLQ6, access to digital services that work; eHLQ7, digital services that suit individual needs), and an understanding of the extent to which users feel supported by relatives, peers, and health professionals (HLQ1, feeling understood and supported by health care providers; HLQ4, social support for health).

Compared with the 2 other instruments we identified that measure readiness, PERQ [10] and SUTAQ [11], READHY is grounded in a modern concept of eHealth literacy (eHealth literacy framework [15]) and addresses users’ knowledge and skills, their self-management of disease, their perceptions and mindset, their experience with health technology systems, and an understanding of the extent to which users feel supported by relatives, peers, and health professionals. PERQ differs by not being conceptually based or psychometrically validated, but rather consists of specific questions in relation to internet use, social support, personal abilities, and economic burden [10]. SUTAQ is, like READHY, conceptually and psychometrically robust. In contrast to READHY, which conceptually builds on eHealth literacy, SUTAQ was developed to assess technology acceptance in the Whole System Demonstrator project [11]. SUTAQ predicts acceptance based on the user’s experience with a given technology, where an experience of reduced privacy and comfort predicts rejection of the technology, whereas an experience of benefits by the users lowers the likelihood of rejection [10]. Compared with READHY, SUTAQ lacks information about the user’s knowledge and skills, as well as the context of the burden of the informant’s condition.

### Evaluation of the Properties of READHY Using Confirmatory Factor Analysis

In our data from a large group of people with a cancer diagnosis engaging in rehabilitation services, we found that discriminant validity between some of the scales was not sufficient, suggesting that a more parsimonious model, with fewer items and scales, might be available. However, we were not able to find a better fit, although our results indicated clear grouping of some dimensions. One example is the high correlation between heiQ3 (self-monitoring and insight) and heiQ5 (skill and technique acquisition). This could be either because the 2 dimensions capture the same construct, or because one (heiQ 5) might *cause* the other (heiQ3); thus, we observed a high correlation between them, but they are different constructs and different interventions would affect changes in these dimensions. Likewise, the connection between eHLQ1, eHLQ3, eHLQ5, eHLQ6, and eHLQ7 may be explained by a causal pathway, that is, having the personal knowledge and skills to engage with digital services and information and having access to systems that work may motivate you to use the systems. While there appears to be some overlap between some of the 13 scales selected, and potential causal hypotheses can be drawn from the data, the 13-factor model works satisfactorily, notwithstanding some factor collapse. If such a pattern is replicated in other patient groups in other settings, particularly among the eHLQ scales, a reduced number of scales might be appropriate for future use, particularly in population studies where questionnaire length may be important. Careful testing of the READHY in other settings receiving technological interventions, such as people with complex conditions such as chronic obstructive pulmonary disease, diabetes, or cardiac heart failure, will provide important empirical information that will assist with reducing the total number of dimensions of the READHY. However, a premature reduction of dimensions may omit information critical in other contexts, such as when robust interventions are introduced, and computer media or system services are changed.

### Cluster Analysis

The combination of hierarchical and k-means cluster analysis is a way of identifying clusters of individuals with respect to their attributes in relation to health technology. We further explored the clusters by adding disease-specific and sociodemographic data (age, smartphone ownership, and number of chronic conditions). These different profiles can be addressed in the form of tailored introductions to technology, educational programs, or variations in user interfaces. In addition, these groups can be used to understand the individual’s attitude and experiences in relation to health technology, their emotional well-being, and the impact of their condition. For a health technology design or service design process, a lower number of clusters would be appropriate, while separating users into further clusters can help understand how different user types can be supported in their use of technology.

The profiles identified in the cluster analysis may be useful for identifying those who are at risk of being marginalized or need particular interventions. We identified such a group in the 8-cluster analysis. In this subgroup (see profile 8, Figure 4), individuals have high emotional distress and may need assistance to learn to cope with and manage their situation to reduce the burden of their condition before being ready to engage with digital services or technologies. Although only 7 individuals belonged to this group, they were likely to be underrepresented in the sample, given the recruitment process, and they represent at least 2% of the population and thus a considerable number of individuals in a national or regional perspective. This group may well be found to be higher users of health care services as their chronic conditions progress [38]. It is important to characterize high-risk groups, which is reflective in important programs such as Denmark’s and other countries’ focus on interventions for the 1% of the population that accounts for almost a third of health care expenses [39-45]. This data-driven approach may be a strong tool to identify particular subpopulations to which specific actions and interventions can be tailored. This approach has been effectively incorporated into health system improvement initiatives such as the OPHELIA process [31,32].

### Strengths and Limitations of the Study

A strength of the study is the way we sought to minimize missing data for people with low literacy, as we ensured that respondents had the opportunity to have the instrument read aloud and filled out for them by a project member. A limitation to this study is the exclusion of ethnic minorities. Furthermore, it has been shown that referral to the Copenhagen Centre for Cancer and Health is not equally distributed by socioeconomic group. Higher educational level is associated with a higher rate of referral to rehabilitation services [46]. Data analysis used a combination of CFA, EFA, and ESEM. While we used confirmatory analyses at the commencement and completion of the sequence, the conclusion that a more parsimonious selection of scales might be possible also relied on information drawn from the exploratory analyses. As such, the analyses should be regarded as principally exploratory, and the final 8-factor solution should be replicated in further studies with different samples.

### Perspectives

With the introduction of READHY, we now have an instrument that can help designers of services and health care providers to better understand an individual’s readiness for using digital health services or technology, not only from the eHealth literacy perspective, but also by including insight into the individual’s social network, including health care providers, and their capability to manage their own health. This is particularly important when digital services are provided to people with 1 or more chronic conditions. For this study, we chose cancer survivors. This group covers a wider age range and includes both sexes. In cancer patients, as well as in other groups of people living with 1 or more chronic conditions, it is important to be aware of the burden of the disease and treatment, and the social support from health care professionals, peers, and relatives to be able to provide the digital services and technology in a way that raises the likelihood that these people will adopt the technology. The development of READHY is the first step. We have submitted data for publication that report on users’ willingness to use technology and more detailed information about the group of cancer survivors (S Rossen MSc PhD, unpublished data, 2018), and a qualitative study exploring individuals’ personal values, perceptions, and experiences in more detail is ongoing. Currently READHY is used in 150 Danish diabetes patients to widen our knowledge about groups living with chronic conditions. READHY is also being used in other studies in Denmark and Norway. In these studies, READHY is being used to understand the segments of users to better develop educative material and strategies, to better design digital solutions that can be tailored to the various segments’ specific needs. We expect that future longitudinal studies will contribute to an understanding of how specific interventions will affect patients’ knowledge and skills, self-management of disease, perceptions and mindset, experience with health technology systems, and social support.

This multidimensional, person-centered evaluation will help designers and providers to address the particular needs identified. Like in the OPHELIA process [31], identification of dimensions that score low in individuals or groups will help to design specific interventions. In this way, READHY can assist in providing more effective and efficient interventions, as they can be developed based on specific gaps or needs.

The READHY instrument, with further testing in a wide range of settings, may be a promising tool to provide technologists, researchers, health care providers, and policy makers with robust information to ensure that fit-for-purpose and inclusive digital health systems are developed and evaluated across health care systems.

### Conclusion

While the READHY is relatively long (13 scales), further efforts to reduce the number of scales seems warranted. While the observed clustering and high correlation between some scales suggests redundancy, many scales are likely to be causally related or influenced by different interventions. Until empirical studies provide evidence on which scales predict future readiness outcomes or respond to specific interventions, we recommend using the current scales or selecting a smaller set based on well-defined a priori hypotheses of which scales are likely to predict target outcomes in new settings. The READHY provides an option for researchers and technology implementers to assess groups of individuals’ readiness for health technology. The data derived from the tool will provide rich information in the form of a situational analysis (prior to implementation) and through the complex transitions that individuals, practitioners, and organizations as a whole go through as technological improvements are applied across the health and social care sectors.

## Appendix

Multimedia Appendix 1 Overview of heiQ dimensions affected during interventions.

Multimedia Appendix 2 Identification of the demarcation point.

Multimedia Appendix 3 Confirmatory factor analysis for the 13-factor model.

Multimedia Appendix 4 Convergent and discriminant validity for the 13-factor model.

Multimedia Appendix 5 Scree plot and parallel analysis for the exploratory factor analysis.

Multimedia Appendix 6 Confirmatory factor analysis for the 8-factor model.

## Abbreviations

CFA: confirmatory factor analysis
CFI: comparative fit index
EFA: exploratory factor analysis
eHLQ: eHealth Literacy Questionnaire
ESEM: exploratory structural equation modeling
heiQ: Health Education Impact Questionnaire
HLQ: Health Literacy Questionnaire
OPHELIA: Optimising Health Literacy and Access
PERQ: Patient eHealth Readiness Questionnaire
READHY: Readiness and Enablement Index for Health Technology
RMSEA: root mean square error of approximation
SUTAQ: Service User Technology Acceptability Questionnaire
TLI: Tucker-Lewis fit index
WLSMV: weighted least squares mean and variance adjusted
