# Supplementary material for: Development of the Multidimensional Readiness and Enablement Index for Health Technology (READHY) Tool to Measure Individuals’ Health Technology Readiness: Initial Testing in a Cancer Rehabilitation Setting
Source: J Med Internet Res. 2019 Feb 12;21(2):e10377. doi: 10.2196/10377 (PMC6404640; doi:10.2196/10377)
Supplement: Multimedia Appendix 1 [file jmir_v21i2e10377_app1.pdf]

Overview of heiQ dimensions that have been reported to change during interventions.

Author, Year	Intervention type	heiQ1	heiQ2	heiQ3	heiQ4	heiQ5	heiQ6	heiQ7	heiQ8
Lee *et al*, 2015 [1]	Health technology			X				X	X
Lee *et al*, 2015 [1]	Health technology	X	X		X	X	X	X	
Lindskrog *et al*, 2017[a]	Health technology					X			X
Laursen et al, 2017 [2]	Health education			X	X	X			X
Holmen et al, 2014 [3]	Health technology					X			
Larsen et al, 2014 [4]	Health technology			X	X	X			

X marks a significant positive change of that dimension in the study. Abbreviations: heiQ1 Health Directed Activity, heiQ2 Positive and Active Engagement in Life, heiQ3 Self-monitoring and Insight, heiQ4 Constructive Attitudes and Approaches, heiQ5 Skill and Technique Acquisition, heiQ6 Social Integration and Support, heiQ 7-Health services Navigation, heiQ8 Emotional distress. [a]personal communication.

1. Lee A, Sandvei M, Hosbond T, Petersen J, Kristiansen KR. Klinisk Integreret Hjemmemonitorering (KIH): Slutrapportering til Fonden for Velfærdsteknologi. MedCom i samarbejde med Region Midtjylland og Region Hovedstaden. [Internet]. 2015. Available from: https://www.digst.dk/Digital-velfaerd/Rapporter-og-analyser/Sundhed/Klinisk-integreret-hjemmemonitorering

2. Laursen DH, Christensen KB, Christensen U, Frølich A. Assessment of short and long-term outcomes of diabetes patient education using the health education impact questionnaire (HeiQ). BMC Res Notes 2017 Jun 15;10(1):213. PMID:28619041

3. Holmen H, Torbjørnsen A, Wahl AK, Jenum AK, Småstuen MC, Årsand E, Ribu L. A Mobile Health Intervention for Self-Management and Lifestyle Change for Persons With Type 2 Diabetes, Part 2: One-Year Results From the Norwegian Randomized Controlled Trial RENEWING HEALTH. JMIR MHealth UHealth 2014 Dec 11;2(4):e57. [doi: 10.2196/mhealth.3882]

4. Larsen MH, Krogstad AL, Aas E, Moum T, Wahl AK. A telephone-based motivational interviewing intervention has positive effects on psoriasis severity and self-management: a randomized controlled trial. Br J Dermatol 2014 Dec;171(6):1458–1469. PMID:25143061
