# Supplementary material for: Development of the Multidimensional Readiness and Enablement Index for Health Technology (READHY) Tool to Measure Individuals’ Health Technology Readiness: Initial Testing in a Cancer Rehabilitation Setting
Source: J Med Internet Res. 2019 Feb 12;21(2):e10377. doi: 10.2196/10377 (PMC6404640; doi:10.2196/10377)

Identification of demarcation point

agglomeration coefficient this step	agglomeration coefficient next step	number of clusters	difference
1388.495	1066.712	2	321.783
1066.712	937.961	3	128.751
937.961	871.813	4	66.148
871.813	807.264	5	64.549
807.264	769.26	6	38.004
769.26	736.711	7	32.549
736.711	704.607	8	32.104
704.607	677.202	9	27.405
677.202	650.835	10	26.367
650.835	629.735	11	21.1
629.735	609.347	12	20.388
609.347	592.824	13	16.523
592.824	576.698	14	16.126
576.698	562.328	15	14.37
562.328	549.536	16	12.792
549.536	537.7	17	11.836
537.7	525.901	18	11.799
525.901	515.288	19	10.613
515.288	505.801	20	9.487
505.801	496.394	21	9.407
496.394	487.019	22	9.375
487.019	477.838	23	9.181
477.838	468.741	24	9.097

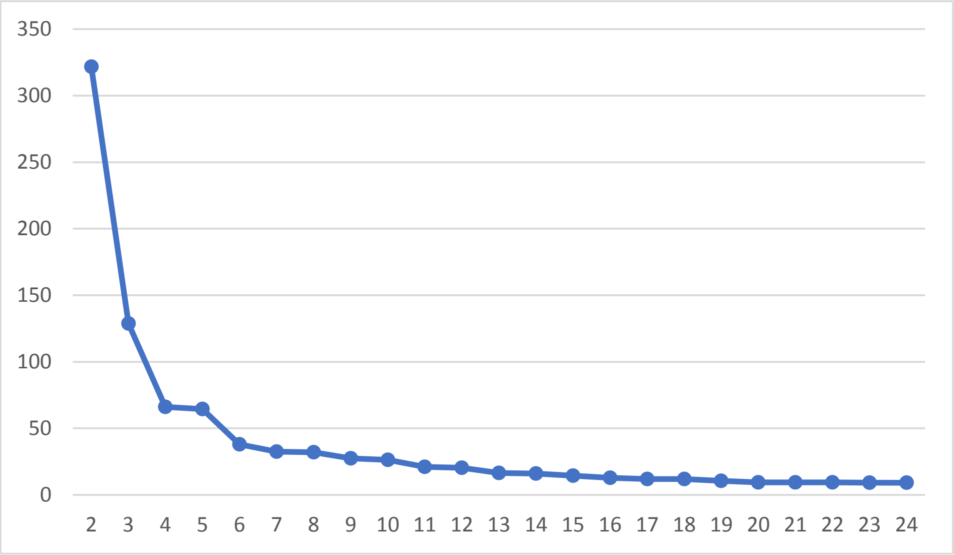

Supplement: Multimedia Appendix 2 [file jmir_v21i2e10377_app2.pdf]
