# Supplementary material for: Development of the Multidimensional Readiness and Enablement Index for Health Technology (READHY) Tool to Measure Individuals’ Health Technology Readiness: Initial Testing in a Cancer Rehabilitation Setting
Source: J Med Internet Res. 2019 Feb 12;21(2):e10377. doi: 10.2196/10377 (PMC6404640; doi:10.2196/10377)
Supplement: Multimedia Appendix 5 [file jmir_v21i2e10377_app5.pdf]

Scree plot

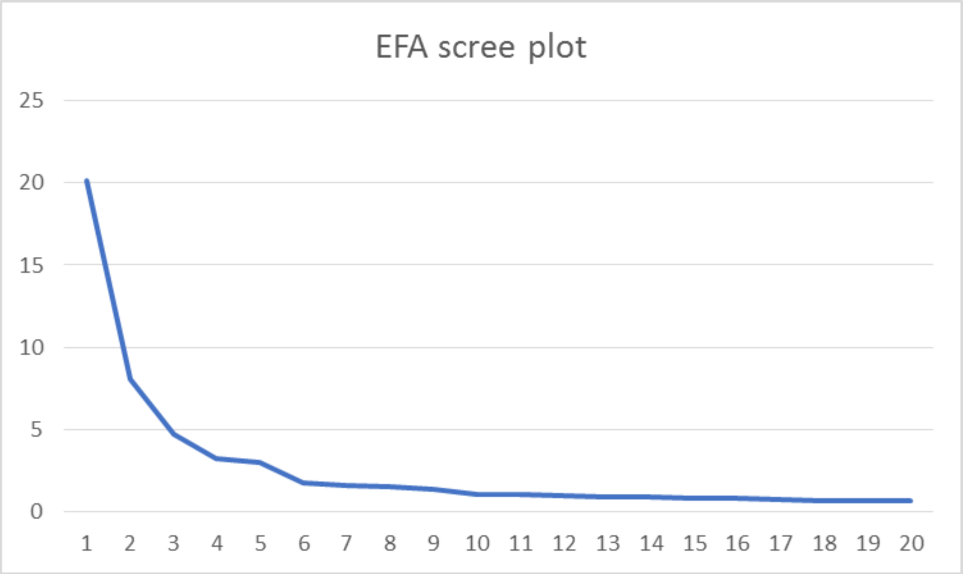

The EFA scree plot suggest 5 or 6 discrete factors.

Parallel analysis

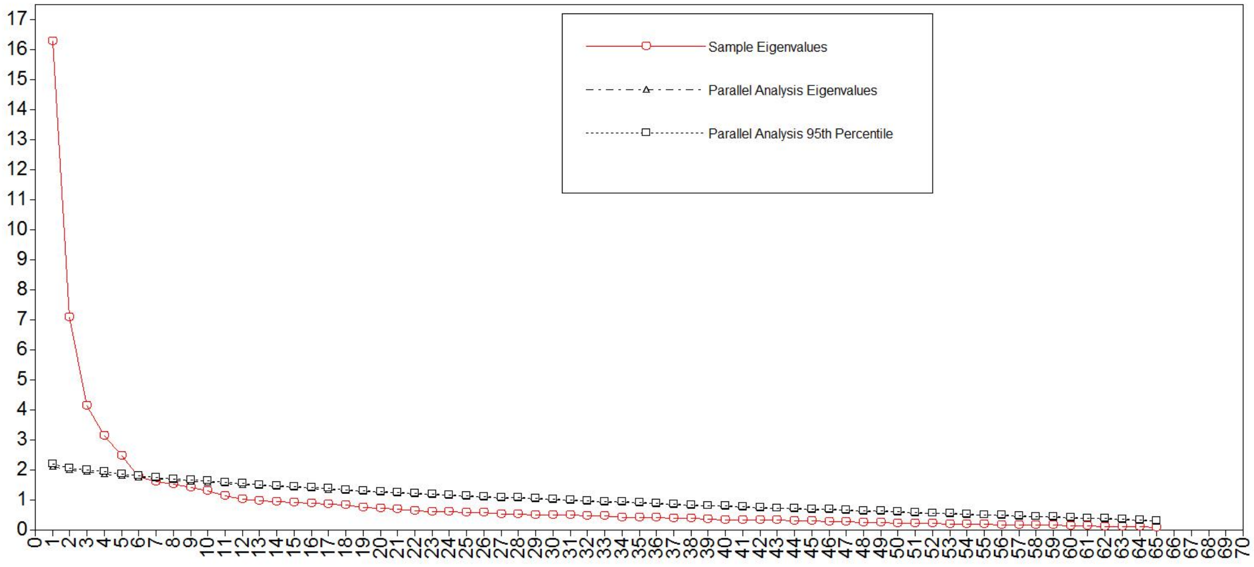

The parallel analysis suggest a 5-factor solution
